# Modulation of HIV Replication in Monocyte-Derived Macrophages (MDM) by Host Antiviral Factors Secretory Leukocyte Protease Inhibitor and Serpin Family C Member 1 Induced by Steroid Hormones

**DOI:** 10.3390/v14010095

**Published:** 2022-01-06

**Authors:** Santanu Biswas, Emily Chen, Yamei Gao, Sherwin Lee, Indira Hewlett, Krishnakumar Devadas

**Affiliations:** 1 Laboratory of Molecular Virology, Division of Emerging and Transfusion Transmitted Diseases, Center for Biologics Evaluation and Research, Food and Drug Administration, 10903 New Hampshire Avenue, Silver Spring, MD 20993-0002, USA; santanu.biswas@fda.hhs.gov (S.B.); emilyy314@gmail.com (E.C.); Sherwin.Lee@fda.hhs.gov (S.L.); 2 Laboratory of Respiratory Viral Diseases, Division of Viral Products, Center for Biologics Evaluation and Research, Food and Drug Administration, 10903 New Hampshire Avenue, Silver Spring, MD 20993-0002, USA; Yamei.Gao@fda.hhs.gov

**Keywords:** antiviral compounds, HIV-1 replication, SLPI, SERPIN C1

## Abstract

The impact of steroid hormones estrogen and progesterone on human immunodeficiency virus type 1 (HIV-1) replication is well documented. However, the exact mechanism involved in the regulation of HIV-1 replication by estrogen and progesterone is still unclear. In the present study, we wanted to elucidate the molecular mechanisms underlying the modulation of HIV-1 replication by estrogen and progesterone. To achieve this goal, we used real-time quantitative PCR arrays (PCR arrays) to identify differentially expressed host genes in response to hormone treatments that are involved in antiviral responses. Our in vitro results suggest that treatment with high doses of estrogen and progesterone promotes the expression of host antiviral factors Secretory leukocyte protease inhibitor (SLPI) and Serpin family C member 1 (SERPIN C1) among others produced in response to HIV-1 infection. SLPI is an enzyme that inhibits human leukocyte elastase, human cathepsin G, human trypsin, neutrophil elastase, and mast cell chymase. SERPIN C1 is a plasma protease inhibitor that regulates the blood coagulation cascade by the inhibition of thrombin and other activated serine proteases of the coagulation system. A dose dependent downmodulation of HIV-1 replication was observed in monocyte-derived macrophages (MDMs) pre-treated with the two proteins SLPI and SERPIN C1. Further investigations suggests that the host antiviral factors, SLPI and SERPIN C1 act at the pre-integration stage, inhibiting HIV-1 viral entry and leading to the observed downmodulation of HIV-1 replication. Our studies would help identify molecular mechanisms and pathways involved in HIV-1 pathogenesis.

## 1. Introduction

The nature of the human immunodeficiency virus (HIV-1) pandemic has changed since its discovery in the 1980s. Continued progress in treatment modalities such as combination antiretroviral therapy (cART) and PrEP, which can effectively control viral replication, has transformed HIV infection from an acute, life-limiting infection to a chronic disease that is manageable with effective treatment. Currently, the reported life expectancy for a newly diagnosed person with HIV-1 on cART is similar to that of the general population but with fewer healthy years [1]. It has also resulted in a remarkable decrease in HIV-1-associated morbidity and mortality.

In addition to the marked changes in life expectancy, the current pattern of HIV-1 transmission is changing, with heterosexual transmission now accounting for greater than 70% of the new cases identified among women in USA [2]. There exists a huge knowledge gap in the identification of molecular mechanisms and pathways leading to sex-based differences in the outcome of HIV-1 infection [3,4]. Early studies on HIV-1 transmission were focused mainly on males and majority of the outcomes and conclusions about pathogenesis, disease progression and therapeutic options were derived from studies conducted in the male population. Numerous epidemiological studies have documented differences between females and males in acquisition rates and manifestations of HIV-1 infection. Recent studies have shown that females are more susceptible to HIV-1 acquisition than males. Following HIV-1 infection, females have lower viral loads during acute infection and exhibit stronger antiviral responses than males, which may contribute to differences in the size of viral reservoirs. Furthermore, immune activation, a hallmark of HIV-1 infection, is generally higher in females than in males and could be a central mechanism in the differences observed in the speed of HIV-1 disease progression in females [5,6]. Several studies have implicated steroid hormones estrogen and progesterone in influencing HIV-1 transmission and modulating HIV-1 replication [7,8,9,10,11,12,13,14,15,16,17]. In regions where young women have the highest worldwide HIV prevalence, the widespread use of progesterone-based injectable hormonal contraceptives may be associated with an immunosuppressive female genital tract environment enhancing the susceptibility to HIV-1 infection [18]. Similarly, several reports have identified steroid hormones estrogen and progesterone as having a profound influence on antiviral innate immune responses that impact the transmission, replication and disease progression during the course of HIV-1 infection [19,20,21,22,23,24,25,26].

HIV-1-infected women experience menopausal symptoms, earlier and in greater intensity. While some data suggest postmenopausal women may be especially vulnerable to HIV-1 acquisition than premenopausal women because of the physiological changes in the vaginal mucosa associated with diminished estrogen levels [27]. As women transition through menopause, hormone therapy (HRT) has been shown to have beneficial effects in certain women, particularly with respect to vasomotor symptoms and bone health. However, in HIV-1-infected post-menopausal women HRT may not be prescribed due to concerns with worsening disease progression and antiretroviral drug toxicities. It is important to understand the effects of altered reproductive hormones on disease progression and response to antiretroviral treatment in HIV-1-infected women [28,29,30].

Although there are several studies that associate steroid hormones and HIV-1 infection, the exact mechanism exerted by estrogen and progesterone in the regulation of HIV-1 transmission and replication is still unclear [31]. In our report, we have investigated the effects of hormones estrogen and progesterone on the replication of HIV-1 in monocyte-derived macrophages. To achieve this goal, we used PCR arrays to identify differentially expressed host genes in response to hormone treatment that are involved in antiviral responses. Our in vitro results suggest that treatment with high doses of estrogen and progesterone promotes the expression of host antiviral factors Secretory leukocyte protease inhibitor (SLPI) and Serpin family C member 1 (SERPIN C1), among others produced in response to HIV-1 infection. SLPI and SERPIN C1 are serpins that belong to a diverse family of protease inhibitors involved in the regulation of physiological processes such as blood clotting, complement activation, programmed cell death, and inflammatory processes [32]. Serpins play an important role in the innate immune response and are part of the early physiologic response to viral infection [32]. Studies suggest that increased levels of serpin expression results in the reduced incidence of HIV acquisition or protracted disease progression in patients [33,34,35]. In addition, several reports have shown that serpins can act as potential inhibitors of HIV-1 replication in the blood and mucosa [31,35,36].

SLPI is an enzyme found in saliva, breast milk and genital secretions that inhibits human leukocyte elastase, human cathepsin G, human trypsin, neutrophil elastase, and mast cell chymase. In addition to its antimicrobial activity, SLPI inhibits HIV-1 infectivity in vitro and is thought to play a critical role in mucosal defense [37]. SLPI present in saliva has been shown to block HIV-1 infection of macrophages and primary T-cells [33,37,38,39,40]. In infants, SLPI present in saliva is shown to reduce mother-to-child HIV-1 transmission through breast milk [41]. SLPI is detected at high concentrations in cervical fluids of exposed but uninfected sex workers [34] and in HIV long-term non-progressors [35,42,43]. SERPIN C1 is a plasma protease inhibitor that regulates the blood coagulation cascade by inhibition of thrombin and other activated serine proteases of the coagulation system. In addition to its potent anti-inflammatory activity, SERPIN C1 is implicated in the inhibition of HIV-1, HCV, HSV-1 and HSV-2 replication [43].

Previously, we showed in an in vitro experiment that HIV-1 replication was modulated in monocyte-derived macrophages (MDMs) treated with different concentrations of steroid hormones estrogen and progesterone. Results demonstrated that high concentrations of estrogen and progesterone down-regulated HIV-1 replication. A greater than two-fold decrease in HIV-1 replication was observed in MDMs infected with HIV-1 BaL pre-treated with 1.75 μM estrogen and with 64 nM progesterone. Results from our previous study suggested that pre-treatment with high doses of estrogen and progesterone down-regulate cytokine and chemokine production and promote an anti-inflammatory environment, which offers protection from the immunopathology associated with HIV-1 infection [30]. However, the exact mechanism exerted by these steroid hormones in the regulation of HIV-1 replication is still unclear. Several studies have implicated steroid hormones in regulating host factor expression and modulating HIV transmission and replication. The immunoregulatory action exerted by estrogen and progesterone treatment is implicated to play an important role in the downmodulation of HIV-1 transmission and replication [44]. Fluctuations in endogenous hormonal levels are also known to alter immunological responses [45,46,47]. Furthermore, changes in the endogenous hormonal levels during the reproductive cycle are known to have an impact on diverse physiological processes such as the secretion of antimicrobial peptides, the regulation of the functional properties of CD8^+^ cells, cytokine secretion and function, secretion and function of chemokines that attract inflammatory cells such as neutrophils, macrophages and natural killer cells [27,47,48,49].

In the current study, a dose dependent downmodulation of HIV-1 replication was observed in monocyte-derived macrophages (MDMs) pre-treated with the two antiviral compounds, SLPI and SERPIN C1. Further investigations suggests that the host antiviral factors, SLPI and SERPIN C1 act at the pre-integration stage inhibiting viral entry leading to the observed downmodulation of HIV-1 replication.

## 2. Materials and Methods

### 2.1. Reagents

17β-estradiol, Cat # E2257 and progesterone, Cat # P7556 were purchased from Sigma Chemical Company, St. Louis, MO, USA. Macrophage colony stimulation factor (M-CSF), Cat # PHC2044 was purchased from Thermo Fisher Scientific, Waltham, MA, USA. SLPI, Cat # 1274-PI-100 and SERPIN C1, Cat # 1267-PI-010 were purchased from R&D Systems, Minneapolis, MN, USA.

### 2.2. Isolation and Culture of Monocyte-Derived Macrophages (MDMs)

Elutriated human monocytes isolated from PBMC were provided by the NIH Blood bank. All donors were seronegative for both HIV-1 and hepatitis B. This study was approved by the NIH ethics committee (study number: 99-CC-0168, PI: Susan F. Leitman, M.D.). Prior to initiation, this study protocol was approved by FDA IRB. Written informed consent was obtained from healthy, normal blood donors for publication of this Case Report and any accompanying images according to the ethical principles of international ethical guidelines for biomedical research involving human subjects. A categorical exemption is in place for CBER/FDA for experimental studies by CBER/FDA researchers using existing, deidentified samples of blood and/or blood products originally obtained under the NIH IRB-approved protocol and consent form 99-CC-0168. The monocytes were differentiated in Dulbecco’s Modified Eagle Medium (DMEM) supplemented with 10% FBS, 100 units/mL Penicillin and 100 μg/mL Streptomycin for 5–7 days in the presence of 0.02 μg/mL macrophage colony stimulation factor (MCSF, Cat#PHC9504, Thermo Fisher, Fredrick, MD, USA) at density of 10^6^ cells/mL. Cells were judged by morphological examination and we found that more than 98% cells were differentiated to macrophages.

### 2.3. Cell Lines and Culture Conditions

The HEK 293T cells, TZM-bl cells, and HeLa cells were grown in DMEM supplemented with 10% fetal bovine serum (Thermo Fisher, Fredrick, MD, USA). TZM-bl is an HIV indicator cell line that measures the level of infectious HIV-1 particles. HeLa cells are human cervical epithelial carcinoma cells (NIH AIDS Research and Reference Reagent Program, Rockville, MD, USA) which lacks CD4 receptor and other HIV-1 specific co-receptors. Transfection of HEK 293T cells was performed with EndoFectin™ Max transfection reagent (Genecopoeia, Rockville, MD, USA) in accordance with the manufacturer’s instructions. The chronically HIV-1-infected U1 cell line was obtained from the NIH AIDS reagent program (NIH, Bethesda) and maintained in suspension culture in RPMI-1640 (Thermo Fisher, Fredrick, MD, USA) supplemented with 10% (*v*/*v*) heat-inactivated fetal bovine serum and 5 µg/mL penicillin/5 µg/mL streptomycin/10 µg/mL neomycin at 37 °C in a humidified atmosphere of 5% CO_2_. U1 cells were induced to activate the HIV-1 virus by treating the cells with 20 ng/mL of phorbol 12-myristate 13-acetate (PMA) for 2 h followed by complete media exchange. Cultures were maintained by half-media exchange every 2 days.

### 2.4. HIV-1 Infection

Primary monocyte-derived macrophages (MDMs) isolated from donors and cultured for 5 days were infected with 5 ng/mL p24 units of CCR-5 tropic HIV-1 BaL(clade B) (Cat# 510, NIH AIDS Reagent, Rockville, MD, USA) [14,50]. After a two-hour exposure, virus particles were removed, and the cells were washed 3 times in 1× PBS. Fresh culture medium was added with appropriate additions of the steroid hormones/SLPI/SERPIN C1 and cells cultured at 37 °C until further use. The following concentrations of SERPIN C1 (0.05 µg/mL and 0.1 µg/mL); SLPI (1 µg/mL and 10 µg/mL); Estrogen: 110 nM, 40 pM; and Progesterone: 64 nM, and 2.5 nM were used. Culture supernatants were collected at specified days post infection and HIV-1 replication quantitated by p24 alphaLISA kits (Perkin Elmer, Boston, MA, USA). After supernatants were harvested, an equivalent volume of growth media containing appropriate concentrations of steroid hormones/SLPI/SERPIN C1 (SERPIN C1 (0.05 µg/mL and 0.1 µg/mL); SLPI (1 µg/mL and 10 µg/mL); Estrogen: 110 nM, 40 pM; and Progesterone: 64 nM, and 2.5 nM) added to each well throughout the experiment.

### 2.5. RNA Isolation and Real-Time Quantitative PCR Array

Total RNA was extracted from samples using the RNeasy plus mini kit (Qiagen, Germantown, MD, USA). Three hundred nanograms of total RNA from each sample was reverse transcribed into cDNA using the RT^2^ first strand kit (Qiagen, Germantown, MD, USA). An RT^2^ Profiler PCR Array (Cat# PAHS-051, Qiagen, Germantown, MD, USA) was used to examine 84 genes which involved in host response to HIV infection. House-keeping genes β-actin, glyceraldehyde 3-phosphate dehydrogenase, β₂ macroglobulin, hypoxanthine phosphoribosyl transferase 1 and ribosomal protein lateral stalk subunit P0 used for normalization. Several negative controls were included in each run. All PCR experiments were conducted with a ViiA 7 Real Time PCR system (Thermo Fisher Scientific, Waltham, MA, USA). Array plates included endogenous controls, reverse transcriptase negative controls, and genomic DNA contamination controls. Ct values of 35 or greater for any particular mRNA in either the control or the experimental samples were excluded from the analysis and marked as undetectable or undetermined. The Ct values that met these stringent criteria were uploaded into the Qiagen software (RT^2^ Profiler PCR Array Data Analysis) and the fold regulation was calculated for each mRNA. Data analysis was performed using the ΔΔCt-based calculations. Assays were performed with RNA samples isolated from MDMs obtained from 3 independent donors.

### 2.6. In Vitro HIV-1 Inhibition Assay

(1) Pre-treatment treatment assay- MDMs (0.5 × 10^6^ cells/well in a 24-well plate) were pre-treated with SERPIN C1 or SLPI and then infected with HIV-1 BaL (5 ng p24/mL/10^6^ cells). After 2 h of virus adsorption, culture medium was replaced with SERPIN C1 (0.05 µg/mL and 0.1 µg/mL) or SLPI (1 µg/mL and 10 µg/mL) containing DMEM culture medium. MDMs were incubated for 7 days at 37 °C under 5% CO_2_.).
(2) Post treatment assay-HIV-1 BaL (5 ng p24/mL/10^6^ cells) infected MDMs (0.5 × 10^6^ cells/well in a 24-well plate) were treated with SERPIN C1 (0.05 µg/mL and 0.1 µg/mL) or SLPI (1 µg/mL and 10 µg/mL) post infection and cultured for 7 days.
(3) Inactivation of HIV-1- HIV-1 BaL (5 ng/mL p24 units) was incubated without or with SLPI (1 µg/mL and 10 µg/mL) or SERPIN C1 (0.05 µg/mL and 0.1 µg/mL) at 37 °C for 2 h in PBS containing 0.1% BSA. After that, MDMs (5 × 10^5^ cells/well) were infected with HIV-1 BaL and incubated at 37 °C for 2 h for virus adsorption washed with phosphate-buffered saline (PBS) and cultured in 2 mL fresh media at 37 °C for 7 days.

### 2.7. HIV-1 Env Pseudo Typed Viral Production and Infection

The plasmid pNL4-3.Luc.R-E- encoding the envelope-deficient HIV-1 NL4-3 virus with the luciferase reporter gene inserted into nef was pseudo typed with the vesicular stomatitis virus envelope protein (VSV-G) by cotransfection with pPACK packaging mix (System Bioscience, Palo Alto, CA, USA) containing pVSV-G in 293T cells using EndoFectin (Genecopoeia, Rockville, MD, USA) in Opti-MEM I medium. Viral particles-containing supernatants were collected after 48 h, cleared of cells and cell debris by sedimentation and filtration as per the manufacturer’s instructions. Viral particles were concentrated by 5× PEG-it solution and quantitated by measuring HIV-1 p24 antigen concentration with the AlphLISA kit (PerkinElmer Life Sciences, Inc., Boston, MA, USA). The extent of HIV-1 entry was determined by an assay based on single-cycle infection. Five to six days before infection, MDMs were seeded into 24-well tissue culture plates at a density of 50,000 per well unless otherwise stated. After 24 h, the cells were differentiated with MCSF. MDMs were pretreated with SERPIN C1 (0.05 µg/mL and 0.1 µg/mL) or SLPI (1 µg/mL and 10 µg/mL) and then the cells were infected with pseudo typed virus (5 ng of p24 antigen) for 2 h at 37 °C, in a total infection volume of 100 µL. Unbound virus was removed by washing, and fresh medium with or without SERPIN C1/SLPI in a total volume of 200 µL was added back to the cells, unless otherwise stated. Seventy-two hours post infection, the cells were washed once with phosphate-buffered saline and the total RNA extracted from samples using the RNeasy plus mini kit (Qiagen, Germantown, MD, USA) and viral RNA expression was quantitated by real-time PCR.

### 2.8. Quantitation of HIV-1 Replication

Culture supernatants were assayed for HIV-1 p24 using an alphLISA p24 analysis kit (Perkin Elmer Life Sciences, Inc., Boston, MA, USA) according to the manufacturer’s instructions. Assays were performed in triplicate. The HIV-1 p24 antigen capture assay cannot differentiate between infectious and noninfectious virus particles. Thus, the measurement of HIV-1 p24 levels does not necessarily reflect the number of infectious units.

### 2.9. SERPIN C1 and SLPI Assay

The concentrations of SERPIN C1 and SLPI in the culture supernatants of MDMs were analyzed with a Bioplex assay kit (Bio-Rad Laboratories, Hercules, CA, USA using a Luminex200 instrument (Luminex Corporation, Austin, TX, USA). The sample concentrations were determined using a Logistic-5PL regression method with the Bio-Plex manager 5.0 software (Bio-Rad Laboratories) according to the manufacture’s protocol.

### 2.10. Detection of Integrated HIV-1 DNA, Early and Late Stage HIV-1 DNA Products

MDMs (0.5 × 10^6^) were treated with SERPIN C1 (0.05 µg/mL and 0.1 µg/mL) or SLPI (1 µg/mL and 10 µg/mL), followed by infection with HIV-1 BaL (5 ng of p24 antigen) for 2 h at 37 °C and unbound virus was removed by washing. As a control, untreated (SERPIN C1/SLPI) cells were used. Cells were collected 16 h after infection, and total DNA extracted using the DNeasy tissue kit (Qiagen, Germantown, MD, USA). Real-time PCR was performed with equal amounts of DNA from different samples using the forward primer 5′-TTAGACCAGATCTGAGCCTGGGAG-3′, reverse primer 5′-GGGTCTGAGGGATCTCTAGTTACC-3′ to amplify the early viral DNA and forward primer 5′-TGTGTGCCCGTCTGTTGTG-3′, reverse primer 5′-GAGTCCTGCGTCGAGA-3′ to amplify the late viral DNA. The reactions were performed with the Fast SYBR™ Green Master Mix (Thermo Fisher, Fredrick, MD, USA) in accordance with the manufacturer’s instructions. The PCR conditions were 95 °C for 10 s, 62 °C for 5 s, and 72 °C for 7 s. In order to measure viral DNA integrated into cellular chromosomal DNA, the first round of PCR was performed with forward primer 5′-GCCTCCCAAAGTGCTGGGATTACAG-3′ and reverse primer 5′-GTTCCTGCTATGTCACTTCC-3′, which binds to Alu and HIV-1 Gag DNA sequences (Alu-gag PCR). The reactions were performed at 94 °C for 1 min to denature DNA templates, followed by 12 cycles at 94 °C for 30 s, 50 °C for 30 s, and 72 °C for 3.3 min. The amplified DNA products were quantified by real-time PCR as described above with the forward primers 5′-TTAAGCCTCAATAAAGCTTGCC-3′, reverse primer 5′-GTTCGGGCGCCA CTGCTAGA-3′, which amplify the HIV-1 long terminal repeat (LTR) region. The HIV-1 2-LTR DNA was amplified with forward primer 5′-CCCTCAGACCCTTTTAGTCAGTG-3′ and the reverse primer 5′-TGGTGTGTAGT TCTGCCAATCA-3′. GAPDH was used as an internal control.

### 2.11. In Trans HIV-1 Transmission Assay

The attachment assay was performed with CD4^−^ CCR5^−^ HeLa cells. HeLa cells (0.05 × 10^6^ cells/well in a 24-well plate) were incubated for 2 h at 37 °C before viral exposure with SERPIN C1 (0.05 µg/mL and 0.1 µg/mL) or SLPI (1 µg/mL and 10 µg/mL). Heparinase I and III (20 IU/mL) were used as negative controls. HIV-1 BaL (10 ng of p24) were added to target cells for 1 h at 37 °C in a final volume of 100 µL of serum free DMEM. Cells were washed five times with 100 µL of PBS to remove unbound material. The HIV-1 indicator cells TZM-bl cells (0.05 × 10^6^ cells/well) were overlaid on to the HeLa cells for 2 h and the medium replaced with fresh DMEM. The attachment assay was monitored sixty hours post infection using the Luciferase Assay System (Promega Inc, Madison, WI, USA). Luciferase activity was measured as per the manufacturer’s instructions.

### 2.12. Statistical Analysis

Non-image statistical analysis was performed using GraphPad Prism 5 software. Error bars were SD or SE from means as indicated. One-way ANOVA with multiple comparisons was used and p values lower than 0.05 were considered significant and higher values were considered not significant (ns).

## 3. Results and Discussion

We have found previously that pretreatment of MDMs with high concentrations of estrogen and progesterone results in the down-regulation of HIV-1 replication and the downmodulation of the expression proinflammatory cytokines and chemokines [31]. However, the exact mechanism involved in the down-regulation of HIV-1 replication by estrogen and progesterone is still unclear. In the present study, we wanted to elucidate the molecular mechanisms underlying the modulation of HIV-1 replication by estrogen and progesterone. Our results suggests that treatment with estrogen and progesterone leads to the up-regulation of host antiviral factors, SLPI and SERPIN C1, which act at the pre-integration stage inhibiting HIV-1 viral entry and the observed downmodulation of HIV-1 replication.

### 3.1. Hormones Induces SERPIN C1 and SLPI in HIV-1-Infected MDMs

In the present study, we wanted to gain further insight into the molecular mechanisms underlying the modulation of HIV-1 replication by estrogen and progesterone. To achieve this goal, we used PCR arrays to identify differentially expressed host factors in response to hormone treatment and HIV-1 infection that are involved in innate immune responses such as CX3CL1, SERPIN C1, SLPI, in apoptosis signaling, in natural killer cell signaling activation pathways, interferon stimulated genes (ISG) including inflammatory cytokines, major chemokines, transcription factors and other host factors.

**Figure 1:**
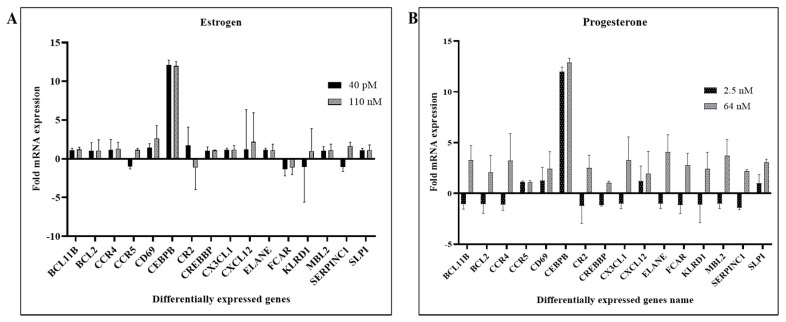
Differentially Expressed Genes in Estrogen or Progesterone Treated MDMs Infected with HIV-1; MDMs (10 × 10 ^6^ cells/flask) were pre-treated with estrogen (40 pM and 110 nM) or progesterone (2.5 nM and 64 nM) and infected with HIV-1 (BaL). After 2 h, the virus was removed, and fresh culture media added with same concentration of estrogen (40 pM and 110 nM) or progesterone (2.5 nM and 64 nM) and cultured. MDMs were collected 9 days post infection. RT^2^ Profiler PCR Array was used to examine the mRNA levels of different HIV-1 host response genes. Assays were performed with experimental RNA samples isolated from MDMs obtained from 3 independent donors. Data was analyzed with average Ct values (Table S1) using Qiagen web-based software (RT^2^ Profiler PCR Array Data Analysis) and results expressed as mean ± SD; (**A**) Differentially expressed genes in estrogen treated MDMs infected with HIV-1 BaL; (**B**) Differentially expressed genes in progesterone treated MDMs infected with HIV-1 BaL.

Using the RT^2^ Profiler PCR Array Data Analysis tool provided by Qiagen, differentially expressed genes were identified based on positive PCR controls and reverse transcription control. In terms of the sensitivity and accuracy, the cutoff fold change (2 − ΔΔCt) was set at greater than 1.5 or less than −1.5. The results indicate that several genes were found to be differentially expressed in MDMs infected with HIV-1 pre-treated with estrogen or progesterone compared to HIV-1-infected MDMs not treated with estrogen or progesterone (Figure 1A,B and Figure S1). The results presented in Figure 1A indicate that in HIV-1-infected cells treated with high concentrations of estrogen (110 nM), certain genes involved in the humoral response, inflammatory response and defensive response against pathogens were up-regulated compared to cells treated with low concentrations of estrogen (40 pM). We have found that SERPIN C1, SLPI and CX3CL1, and other host response genes such as CD69 and CXCL12 were up-regulated in HIV-1-infected cells treated with estrogen. On the other hand, presented in Figure 1B, treatment with high concentration of progesterone (64 nM) resulted in the up-regulation of innate immune responsive genes, inflammatory genes, defensive response against virus related genes, transcription factors and host response genes. Specifically, genes such as CX3CL1, SERPIN C1, SLPI, and transcription factor BCL11B were up-regulated only in HIV-1-infected cells treated with high concentration of progesterone. The CCAAT/enhancer binding protein beta (CEBPB) gene was dramatically up-regulated by both the hormones in HIV-1-infected MDMs. Similarly, others have also found that the CEBPB gene is positively correlated with HIV infection [51]. There were no changes in gene expression pattern (Table S2) in uninfected cells treated with estrogen or progesterone, implying that estrogen or progesterone treatment synergistically modulates the expression of host responsive genes in response to HIV-1 infection.

**Figure 2:**
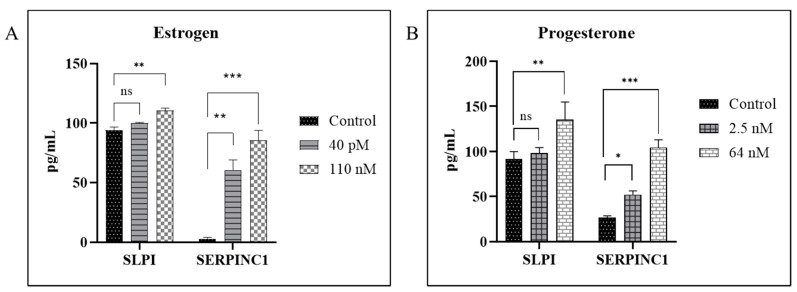
Validation of Protein expression in Estrogen or Progesterone Treated MDMs Infected with HIV-1; BioPlex analysis of secreted Secretory Leukocyte Peptidase Inhibitor (SLPI) and Serine Proteinase Inhibitor Clade C Member 1 (SERPIN C1) in culture supernatants from monocytes derived macrophage cells infected with HIV-1 (BaL) pre-treated with 40 pM or 110 nM estrogen (**A**) or 2.5 nM or 64 nM progesterone (**B**) and not pre-treated with estrogen or progesterone as control. Culture supernatants were analyzed in duplicate; Culture supernatants were analyzed in triplicate. Results expressed as mean ± SEM. This data is representative of three independent donors. Asterisk (*) over the bars indicates significant difference with control; *** *p* < 0.0001; ** *p* < 0.001; * *p* ≤ 0.05 and ns *p* > 0.05. *p*-values were generated by one-way ANOVA with multiple comparisons.

To validate the differentially expressed host responsive genes in response to estrogen and progesterone treatment identified by the RT^2^ PCR array we quantitated the expression levels of SERPIN C1 and SLPI at the protein level, in culture supernatants from HIV-1-infected cells treated with 110 nM estrogen (high concentration) and 40 pM estrogen (low concentration) or 64 nM progesterone (high concentration) and 2.5 nM progesterone (low concentration). Our results (Figure 2A, Figures S2A and S3A) indicate that the levels of SERPIN C1 and SLPI were up-regulated in culture supernatants collected from HIV-1-infected MDMs treated with estrogen (110 nM and 40pM) compared to untreated HIV-1-infected cells. Similarly, results (Figure 2B, Figures S2B and S3B) indicate that the levels of, SERPIN C1 and SLPI were up-regulated in culture supernatants collected from HIV-1-infected MDMs treated with high concentrations of progesterone (64 nM) compared to untreated HIV-1-infected cells and HIV-1-infected MDMs treated with low concentrations of progesterone (2.5 nM).

### 3.2. Human Recombinant Protein SERPIN C1 and SLPI Modulates HIV-1 Replication

**Figure 3:**
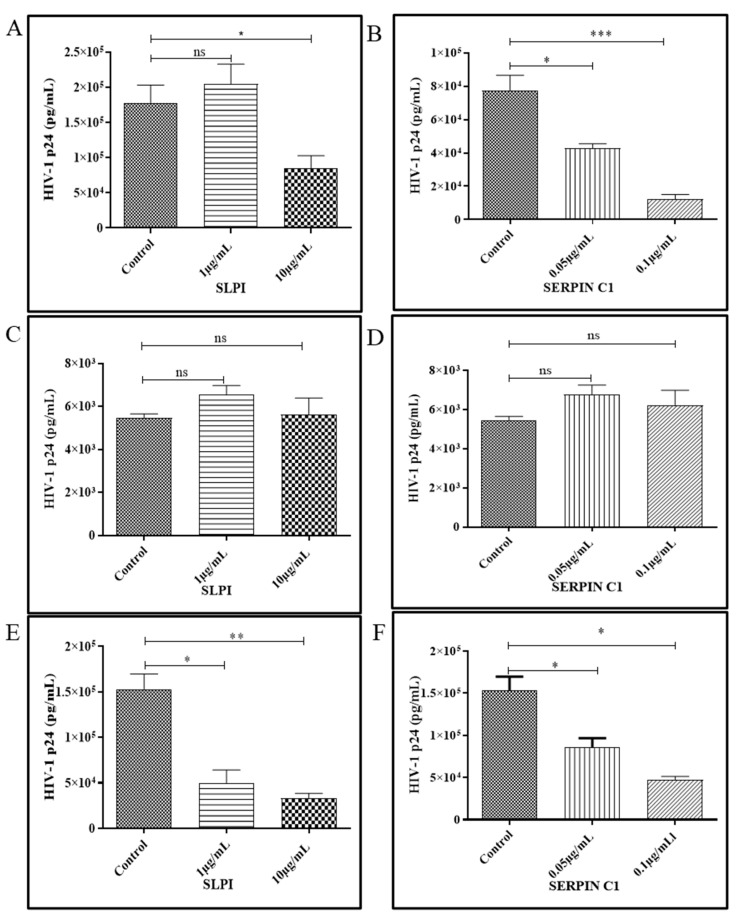
HIV-1 Replication in Pre-treated MDMs (**A**,**B**), Post HIV-1-infected MDMs (**C**,**D**) and -MDMs infected with pre-incubated inoculum- (**E**,**F**) treated with SLPI and SERPINC1. (**A**,**B**) MDMs (1 × 10^6^ cells/well) were pre-treated with (**A**) SLPI 1 µg/mL and 10 µg/mL or (**B**) SERPIN C1 0.05 µg/mL and 0.1 µg/mL concentration for 3 h then infected with HIV-1 BaL 5 ng/mL p24 units. After 2 h the virus was removed, and fresh culture media added with 1 µg/mL and 10 µg/mL SLPI or 0.05 µg/mL and 0.1 µg/mL SERPINC1 and cultured. (**C**,**D**) MDMs (1 × 10^6^ cells/well) were infected with HIV-1 BaL 5 ng/mL p24 units. After 2 h, the virus was removed, and fresh culture media added with (**C**) SLPI 1 µg/mL and 10 µg/mL concentration or (**D**) SERPIN C1 0.05 µg/mL and 0.1 µg/mL and cultured the MDMs. (**E**,**F**) HIV-1 BaL (5 ng/mL p24 units) was incubated without or with (**E**) SLPI 1 µg/mL and 10 µg/mL or (**F**) SERPIN C1 0.05 µg/mL and 0.1 µg/mL at 37 °C for 2 h in PBS containing 0.1% BSA. After that, MDMs (5 × 10^5^ cells/well) were infected with HIV-1 BaL and incubated at 37 °C for 2 h for virus adsorption washed with phosphate-buffered saline (PBS) and cultured in 2 mL fresh media at 37 °C. Culture supernatants were collected after 7 days post infection and HIV-1 replication quantitated by HIV-1 p24 ELISA. Culture supernatants were analyzed in triplicate. Results expressed as mean ± SEM. This data is representative of three independent donors. Asterisk (*) over the bars indicates significant difference with control, *** *p* < 0.0001; ** *p* < 0.001; * *p* ≤ 0.05 and ns *p* > 0.05. *p*-values were generated by one-way ANOVA with multiple comparisons**.**

In addition to its potent anti-inflammatory activity, SERPIN C1 is implicated in the inhibition of HIV-1, HCV, HSV-1 and HSV-2 replication [41]. SLPI has been described as an important HIV-1 inhibitory factor in saliva that acts as a potent suppressor of HIV-1 infection [30,37]. To determine if pre-treatment with SLPI and SERPIN C1 modulates HIV-1 replication, MDMs were treated with SLPI (10 µg/mL and 1 µg/mL) and SERPIN C1 (0.1 µg/mL and 0.05 µg/mL) for two hours prior to infection and infected with R5-tropic HIV-1 BaL. After 2 h incubation, MDMs were extensively washed to remove residual input virus and cultured for 7 days. After washing (2 h post infection), SERPIN C1 or SLPI was added back to the culture media. The culture media was replenished every three days. Untreated MDMs were included as controls. Cell culture supernatants were collected on day 7 post infection and HIV-1 p24 levels were quantitated. Results from these experiments demonstrated a dose dependent decrease in HIV-1 p24 levels in supernatants from MDMs treated with SERPIN C1 compared to untreated control cells at day 7 post infection. Greater than six-fold decrease (84% decrease) in HIV-1 replication was observed in MDMs infected with HIV-1 BaL pre-treated with 0.1 µg/mL SERPIN C1 and 45% down-regulation of HIV-1 replication was observed in MDMs infected with HIV-1 BaL pre-treated with 0.05 µg/mL SERPIN C1 (Figure 3B, Figures S4B and S5B). In MDMs, pre-treatment with SLPI at high concentrations (10 µg/mL) near about 50% reduction in virus replication was observed compared to untreated HIV-1-infected MDMs (Figure 3A, Figures S4B and S5B). We next investigated the effect of SLPI and SERPIN C1 treatment after MDMs were infected with HIV-1. MDMs were first incubated with HIV-1 for 2 h, and extensively washed to remove residual input virus. After washing (2 h post infection), SLPI (10 µg/mL and 1 µg/mL) and SERPIN C1 (0.1 µg/mL and 0.05 µg/mL) were added to the culture media and replenished every three days. Untreated MDMs were included as controls. After day 7 post infection, HIV-1 p24 antigen was measured to quantitate HIV-1 replication in MDMs. Addition of SLPI or SERPINC1 post infection had no effect on HIV-1 replication in MDMs (Figure 3C,D, Figures S4C,D and S5C,D). To determine whether SLPI and SERPIN C1 physically interacts with HIV-1 and decreases infectivity, HIV-1 BaL was incubated with SLPI (10 µg/mL and 1 µg/mL) or SERPIN C1 (0.1 µg/mL and 0.05 µg/mL) for 2 h prior to infecting MDMs. Untreated HIV-1 BaL was used a control. After day 7 post infection, HIV-1 p24 was quantitated in culture supernatants. The results demonstrate (Figure 3E,F, Figures S4E,F and S5E,F) that incubating HIV-1 BaL with SLPI and SERPIN C1 prior to infection causes a dose dependent down-regulation of HIV-1 replication. Treatment with high concentration of SLPI and SERPIN C1 showed more that 50% inhibition of HIV-1 replication compared to the untreated HIV-1 BaL. Together, these data suggest that when MDMs or HIV-1 were pre-treated with SLPI and SERPIN C1 HIV-1 replication was inhibited. However, there was no effect of SERPIN C1 or SLPI treatment on HIV-1 replication post infection.

The impact of pre-treatment of MDMs with SERPIN C1 or SLPI on cell viability was evaluated using the alamarBlue cell viability assay and the integrity of cell membrane was evaluated by measuring the amount of LDH released from the cells after 48 h of exposure to increased concentration of SERPIN C1 from 0.001 µg/mL to 0.1 µg/mL and for SLPI from 0.01 µg/mL to 10 µg/mL. Our results (data not shown) indicate that pre-treatment with SERPIN C1 and SLPI did not affect MDM viability or cytoplasmic membrane integri-ty up to 0.1 µg/mL and 10 µg/mL, respectively.

### 3.3. SERPIN C1 or SLPI Reduces the Level of Integrated HIV-1 DNA

We next investigated which step of HIV-1 infection is impacted by SERPIN C1 or SLPI pre-treatment. We first monitored the early events of HIV-1 infection by performing real-time PCR to quantify the products of HIV-1 reverse transcription as well as viral DNA integration in the infected MDMs. For each experiment, we calculated the ratios of viral DNA amounts produced in untreated control cells versus those treated with SERPIN C1 or SLPI.

**Figure 4:**
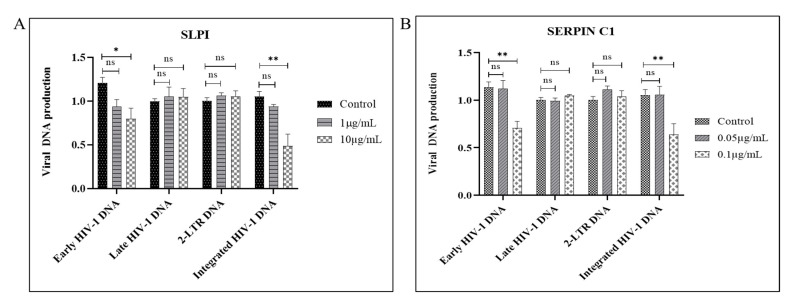
Modulation of early and integrated HIV-1 DNA production by SLPI (**A**) or SERPIN C1 (**B**); MDMs (5 × 10^5^ cells/well) were pre-treated with (**A**) SLPI 1 µg/mL and 10 µg/mL or (**B**) SERPIN C1 0.05 µg/mL and 0.1 µg/mL for 3 h then infected with HIV-1 BaL 5 ng/mL p24 units. After 2 h, the virus was removed, and fresh culture media added with 1 µg/mL and 10 µg/mL SLPI or 0.05 µg/mL and 0.1 µg/mL SERPIN C1 and cultured. MDMs were collected 16 h after infection, and total DNA was extracted with the DNeasy tissue kit. Real-time PCR was performed with equal amounts of DNA and measured the amounts of early and late reverse transcription DNA products, as well as 2-LTR circle DNA. Levels of integrated viral DNA were measured by Alu-PCR. Effect of SLPI and SERPIN C1 on HIV-1 DNA production was determined by calculating the ratios of viral DNA amounts produced without SLPI or SERPIN C1 versus treatment with SLPI or SERPIN C1 in MDMs. Results expressed as mean ± SEM from three independent donors. Asterisk (*) over the bars indicates significant difference with control, ** *p* < 0.001; * *p* ≤ 0.05 and ns *p* > 0.05. *p*-values were generated by one-way ANOVA with multiple comparisons.

Results showed that the level of early HIV-1 DNA product was reduced by pre-treatment with high concentrations of SERPIN C1 or SLPI (Figure 4A,B). Data showed that in high concentration (0.1 µg/mL) SERPIN C1 pre-treated MDMs, a 40% decrease in the early-stage HIV-1 viral DNA production was observed compared to untreated MDMs, whereas in MDMs infected with HIV-1 BaL pre-treated with 10 µg/mL SLPI, a 42% down-regulation of early HIV-1 DNA production compared with HIV-1 BaL infected untreated MDMs was observed (Figure 4A,B).

Next, we evaluated late-stage HIV-1 DNA production and found (Figure 4A,B) that the final stage of HIV-1 DNA production was not impacted by SERPIN C1 or SLPI treatment. These results taken together suggests that pre-treatment with these two compounds leads to a reduction in early HIV-1 DNA or minus strand strong stop DNA synthesis. Previously, it was shown that at least two early HIV-1 DNA transfer reactions (PCR minus strand transfer) were needed to produce a full-length copy DNA of the retroviral RNA genome [52]. Other groups have also shown that two DNA strand transfer reactions occur during retroviral reverse transcription [53]. Thus, our results indicate that the number of starting HIV-1 RNA molecules was less due to an inhibition of virus attachment or entry caused by the pre-treatment with SERPIN C1 or SLPI.

We have also measured the level of viral 2-long terminal repeat (LTR) circle DNA that is only formed within the nucleus and has thus been utilized as a marker to evaluate the nuclear entry of HIV-1 DNA [54]. No significant change was detected by either SERPIN C1 or SLPI pre-treatment (Figure 4A,B), indicating that pre-treatment has no impact on nuclear entry.

Next, we measured the levels of the integrated HIV-1 DNA after pre-treatment with SERPIN C1 or SLPI. Our results indicate that a significant reduction in integrated HIV-1 DNA copies, with a mean decrease of 44% in MDMs treated with high concentrations (0.1 µg/mL) of SERPIN C1 compared to control was detected (Figure 4B) and in MDMs pre-treated with high concentration (10 µg/mL) of SLPI a substantially greater level of reduction, a 52% reduction in integrated HIV-1 DNA was detected (Figure 4A). Taken together, all the results suggest that SERPIN C1 or SLPI pre-treatment impacted HIV-1 DNA production and impairs the integration of HIV-1 DNA into the host DNA. Our results are consistent with reports indicating that the inhibition of HIV-1 by SLPI occurs prior to viral transcription [36].

### 3.4. Impact of SERPIN C1 or SLPI on HIV-1 Replication Post Integration

**Figure 5:**
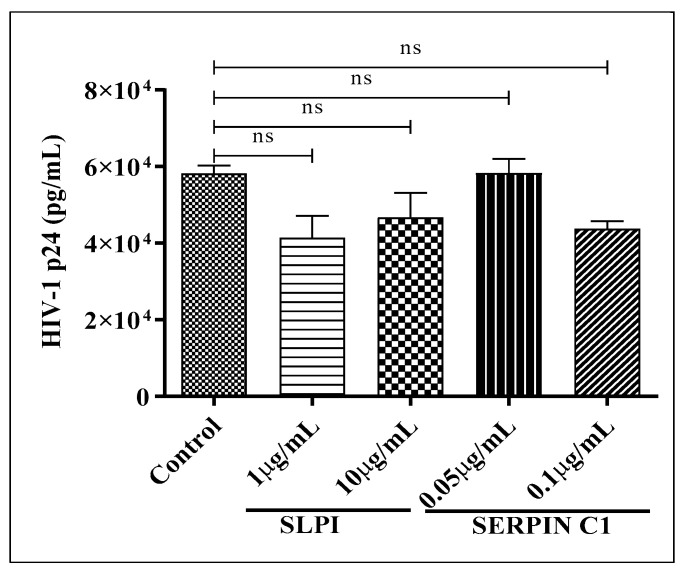
Impact of SLPI and SERPIN C1 on HIV-1 replication post integration. U1 cells (5 × 10^5^ cells/well) were pre-treated with SLPI (1 µg/mL and 10 µg/mL) or SERPIN C1 (0.05 µg/mL and 0.1 µg/mL) for 2 h followed by activation by PMA (20 ng/mL) for 48 h. Culture supernatants were collected and HIV-1 p24 antigen was quantitated by ELISA. Results expressed as mean ± SEM from three independent experiments. Mark (ns) over the bars indicates the non-significant difference with control, ns *p* > 0.05. *p*-values were generated by one-way ANOVA with multiple comparisons.

To further determine that treatment with SERPIN C1 or SLPI has no effect on HIV-1 replication post integration, we used U1 cells that contain replication competent copies of HIV-1 DNA integrated into the host genome. In this experiment, we pre-treated U1 cells with appropriate concentrations of SLPI (10 µg/mL and 1 µg/mL) and SERPIN C1 (0.1 µg/mL and 0.05 µg/mL) for 2 h, followed by activation by PMA for 48 h. After 48 h, HIV-1 p24 antigen was quantitated in culture supernatants. The results indicated that no statistically significant reduction in HIV-1 p24 antigen production was detected (Figure 5). These results (Figure 5) along with the previous results (Figure 3C,D) suggests that SERPIN C1 or SLPI have no effect on the modulation of HIV-1 replication once HIV-1 DNA is integrated into the host genome.

### 3.5. SERPIN C1 or SLPI Impede HIV-1 Entry into Cells

**Figure 6:**
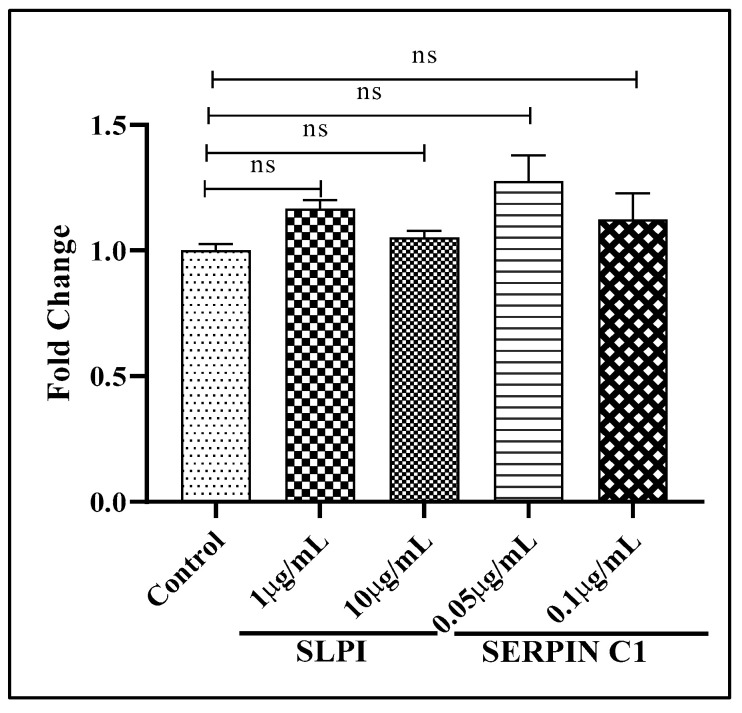
Effects of SLPI and SERPIN C1 on MDMs infection by VSV-G pseudo typed HIV-1 construct.; MDMs (5 × 10^4^ cells/well) were pre-treated with 1 µg/mL and 10 µg/mL SLPI or 0.05 µg/mL and 0.1 µg/mL SERPIN C1 for 2 h and then infected with VSV-G pseudo typed HIV-1 (5 ng/mL p24 units). After 2 h, the unbound virus was removed and replenished with medium containing the same concentrations of SLPI (1 µg/mL and 10 µg/mL) or SERPIN C1 (0.05 µg/mL and 0.1 µg/mL). MDMs were collected 72 h after infection, and total RNA extracted, and real-time PCR was performed to quantitate HIV-1 replication. Results expressed as mean ± SEM from three independent donors. Mark over the bars indicates non-significant difference with control, ns *p* > 0.05. *p*-values were generated by one-way ANOVA with multiple comparisons.

Our results implied that pre-treatment with high concentrations of SLPI and SERPIN C1 inhibits HIV-1 entry into MDMs. However, based on reports in the literature, it seems unlikely that pre-treatment with SERPIN C1 or SLPI directly inhibits the interaction of HIV-1 with the CD4 receptor and CCR5 co-receptor [32,34,37,40]. To confirm whether pre-treatment with SLPI and SERPIN C1 plays a role in inhibiting HIV-1 interactions, with CD4/CCR5 co-receptors effectively preventing co-receptor-mediated HIV-1 entry into MDMs, and that pre-treatment with SERPIN C1 or SLPI has no impact on viral replication post integration, we used a pseudo-typed HIV-1 construct in which the HIV-1 envelope was replaced with the envelope of vesicular stomatitis virus (VSV). The VSV-G pseudo typed virus does not possess HIV-1 envelope glycoproteins and can enter macrophages through a CD4/CCR5-independent pathway and is able to complete only a single round of infection, thus preventing multiple rounds of infection. HIV-1 viral RNA production in MDMs infected with the VSV-G pseudo typed HIV-1 virus was quantitated by RT-PCR. The results indicate that pre-treatment with SLPI and SERPIN C1 had no impact on the replication of the VSV-G pseudo-typed virus (Figure 6). Therefore, it appears that pre-treatment with SLPI and SERPIN C1 has no effect on CD4/CCR5 receptor independent entry and subsequent replication but acts by blocking the interaction of HIV-1 with CD4/CCR5 coreceptors inhibiting viral entry into MDMs.

**Figure 7:**
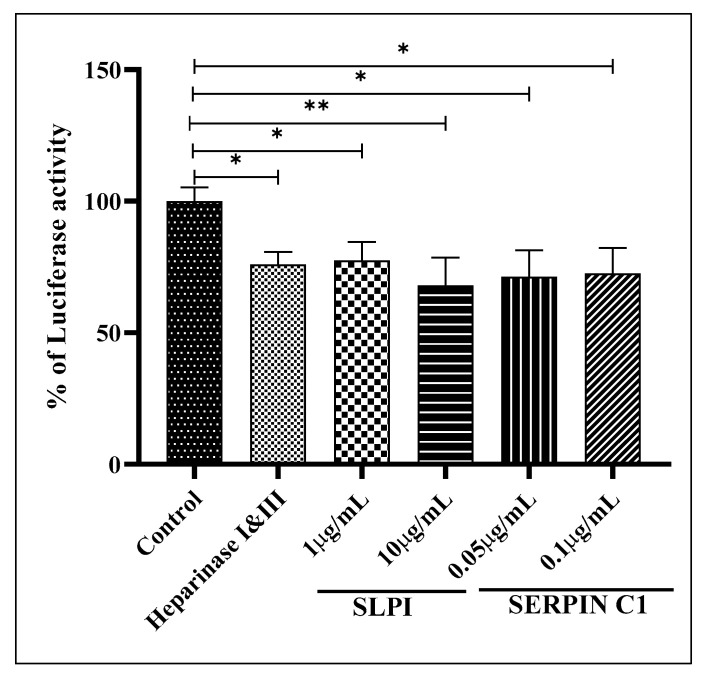
In Trans HIV-1 transmission Assay; HeLa cells (0.05 × 10^6^ cells/well in a 24-well plate) were incubated for 2 h at 37 °C with SLPI (1 µg/mL and 10 µg/mL) or SERPIN C1 (with 0.05 µg/mL and 0.1 µg/mL). Heparinase I and III (20 IU/mL) was used as a negative control. HIV-1 BaL (10 ng of p24) was added to HeLa cells target cells for 1 h at 37 °C in a final volume of 100 µL of serum free DMEM to facilitate virus adsorption. Cells were washed five times with 100 µL of PBS to remove unbound material and overlaid with TZM-bl indicator cells (0.05 × 10^6^ cells/well). After sixty hours post infection productive HIV-1infection and replication was quantitated using the Luciferase Assay System. Results are expressed as mean ± SEM from three independent experiments. Asterisk (*) over the bars indicates significant difference with control, ** *p* < 0.001 and * *p* ≤ 0.05. *p*-values were generated by one-way ANOVA with multiple comparisons.

While CD4 and CCR5 act as the receptor and co-receptor for HIV-1 entry into macrophages, reports suggest that other cell surface molecules such as Heparan sulfate proteoglycans (HSPGs) may serve as an attachment receptor and facilitate HIV-1 entry into MDMs [55,56,57,58,59,60]. Especially since MDMs are known to express low levels of CD4 compared to HSPGs, the initial attachment of HIV-1 to HSPGs may represent a crucial rate limiting step in HIV-1 entry [57,58]. SERPIN C1 and SLPI are also known to bind to the syndecan family of HSPGs [61,62,63]. Thus, taken together, it is likely that SERPIN C1 or SLPI pre-treatment interferes with the initial attachment of HIV-1 to HSPGs and inhibits entry into MDMs. To determine if HSPGs are disrupted by SLPI and SERPIN C1 pre-treatment thus preventing HIV-1 attachment and entry into host cells, we used non-permissive CD4^−^ CCR5^−^ HeLa cells. CD4^−^ CCR5^−^ HeLa cells were pre-treated with SLPI and SERPIN C1 to evaluate HIV-1 attachment and transmission to permissive indicator TZM-bl-cells. CD4^−^ CCR5^−^ HeLa cells treated with Heparinase I and III were used as negative controls. Our results indicate that the non-permissive CD4^−^ CCR5^−^ HeLa cells could efficiently bind and transmit HIV-1 to the permissive indicator TZM-bl-cells (Figure 7). In contrast, the capacity of CD4^−^ CCR5^−^ HeLa cells to attach HIV-1 was low in cells pre-treated with SLPI and SERPIN C1 when compared to the untreated control cells and was comparable to that of Heparinase I- and III-treated cells (Figure 7). These results indicate that SLPI and SERPIN C1 inhibit in trans HIV-1transmission into the cells by disrupting the binding of HIV-1 to HSPGs.

## 4. Conclusions

Several studies have identified steroid hormones estrogen and progesterone as having a profound influence on anti-viral innate immune responses that impact the transmission, replication and disease progression during the course of HIV-1 infection. However, the exact mechanism involved in the regulation of HIV-1 replication by estrogen and progesterone is still unclear. In the present study, we wanted to elucidate the molecular mechanisms underlying the modulation of HIV-1 replication by estrogen and progesterone. To achieve this goal, we used PCR arrays to identify differentially expressed host genes in response to hormone treatment that are involved in antiviral responses. Our in vitro results suggest that treatment with high doses of estrogen and progesterone promotes the expression of host antiviral factors Secretory leukocyte protease inhibitor (SLPI) and Serpin family C member 1 (SERPIN C1) among others produced in response to HIV-I infection. A dose-dependent downmodulation of HIV-1 replication was observed in monocyte-derived macrophages (MDMs) pre-treated with the two compounds, SLPI and SERPIN C1. Further investigations suggests that the host antiviral proteins, SLPI and SERPIN C1, act at the pre-integration stage by blocking HIV-1 binding to HSPGs, preventing trans transmission-mediated CD4/CCR5 transfer into MDMs and leading to the observed downmodulation of HIV-1 replication.

## Supplementary Materials

The following supporting information can be downloaded at: https://www.mdpi.com/article/10.3390/v14010095/s1, Figure S1: Volcano plots of significant gene, expression fold change for estrogen or progesterone treated HIV-1 infected MDMs in comparison to untreated HIV-1 infected MDMs as control; Figure S2: Validation of Protein expression in Estrogen or Progesterone Treated MDMs Infected with HIV-1 (Donor 2); Figure S3: Validation of Protein expression in Estrogen or Progesterone Treated MDMs Infected with HIV-1 (Donor 3); Figure S4: HIV-1 Replication in Pre-treated MDMs (A & B), Post HIV-1 infected MDMs (C & D) and -MDMs infected with pre-incubated inoculum- (E & F) treated with SLPI and SERPINC1 (Donor 2); Figure S5: HIV-1 Replication in Pre-treated MDMs (A & B), Post HIV-1 infected MDMs (C & D) and -MDMs infected with pre-incubated inoculum- (E & F) treated with SLPI and SERPINC1 (Donor 3); ; Table S1: Average Ct values of host genes expressed in Estrogen or Progesterone Treated MDMs Infected with HIV-1; Table S2: Fold Regulation values of host genes expressed in Estrogen or Progesterone Treated MDMs compared with untreated MDMs.

## Additional Information

This article reflects the views of the authors and should not be construed to represent FDA’s views or policies.
