# Supplementary material for: Modulation of HIV Replication in Monocyte-Derived Macrophages (MDM) by Host Antiviral Factors Secretory Leukocyte Protease Inhibitor and Serpin Family C Member 1 Induced by Steroid Hormones
Source: Viruses. 2022 Jan 6;14(1):95. doi: 10.3390/v14010095 (PMC8777669; doi:10.3390/v14010095)
Supplement: Supplementary file 1 [file viruses-14-00095-s001.zip › Figure S1-S5.pdf]

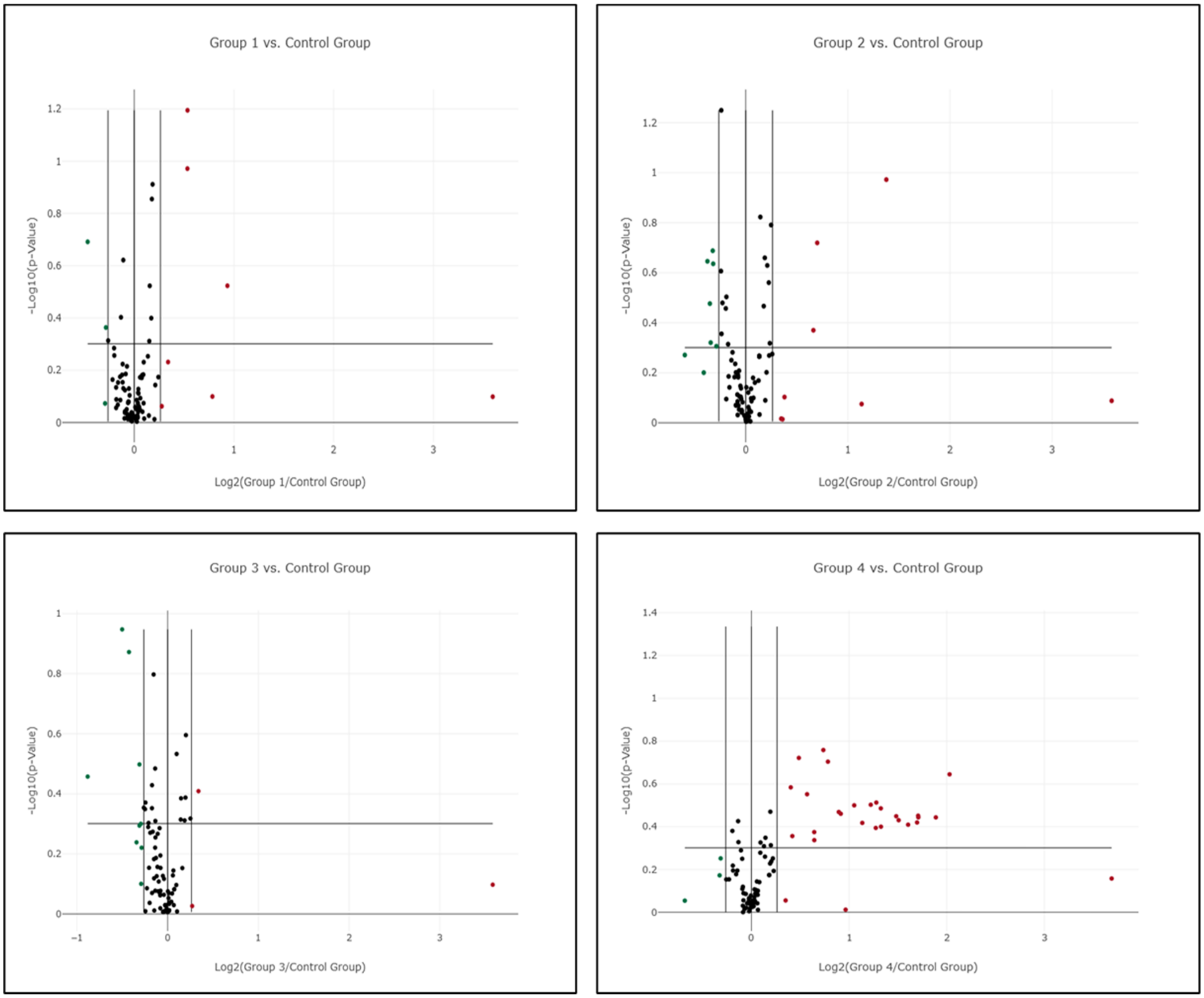

Figure S1: Volcano plots of significant gene, expression fold change for estrogen or progesterone treated HIV-1 infected MDMs in comparison to untreated HIV-1 infected MDMs as control: The plot shows differentially regulated gens with statistical significance that passed Volcano Plot filtering (Fold Change ≥ 1.2 and p-value ≤ 0. 5). The Volcano Plot identifies significant gene expression changes by plotting the log2 of the fold changes in gene expression on the x-axis versus their statistical significance on the y-axis. The center vertical line indicates unchanged gene expression, while the two outer vertical lines indicate the selected fold regulation threshold. The horizontal line indicates the selected p-value threshold. The red point in the plot represents up regulated genes and green point in the plot represents down regulated genes with statistical significance (≤ 0. 5).
Group 1 represented as 40pM estrogen treated MDMs infected with HIV-1
Group 2 represented as 110nM estrogen treated MDMs infected with HIV-1
Group 3 represented as 2.5nM progesterone treated MDMs infected with HIV-1
Group 3 represented as 64nM progesterone treated MDMs infected with HIV-1
Control group represent as MDMs infected with HIV-1

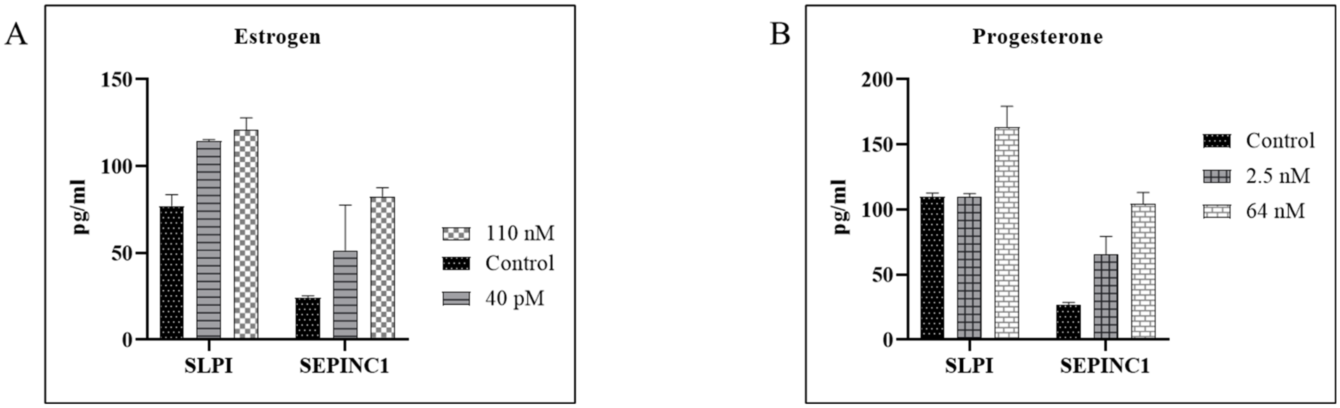

Figure S2: Validation of Protein expression in Estrogen or Progesterone Treated MDMs Infected with HIV-1 (Donor 2).
(A). BioPlex analysis of secreted Secretory Leukocyte Peptidase Inhibitor (SLPI) and Serine Proteinase Inhibitor Clade C Member 1 (SERPIN C1) in culture supernatants from monocytes derived macrophage cells infected with HIV-1 (BaL) pre-treated with 40 pM or 110 nM estrogen (A) or 2.5 nM or 64 nM progesterone (B) and not pre-treated with estrogen or progesterone as control. Culture supernatants were analyzed in duplicate.
Culture supernatants were analyzed in triplicate. Results expressed as mean ± SEM of three independent experimental set up of Donor 2

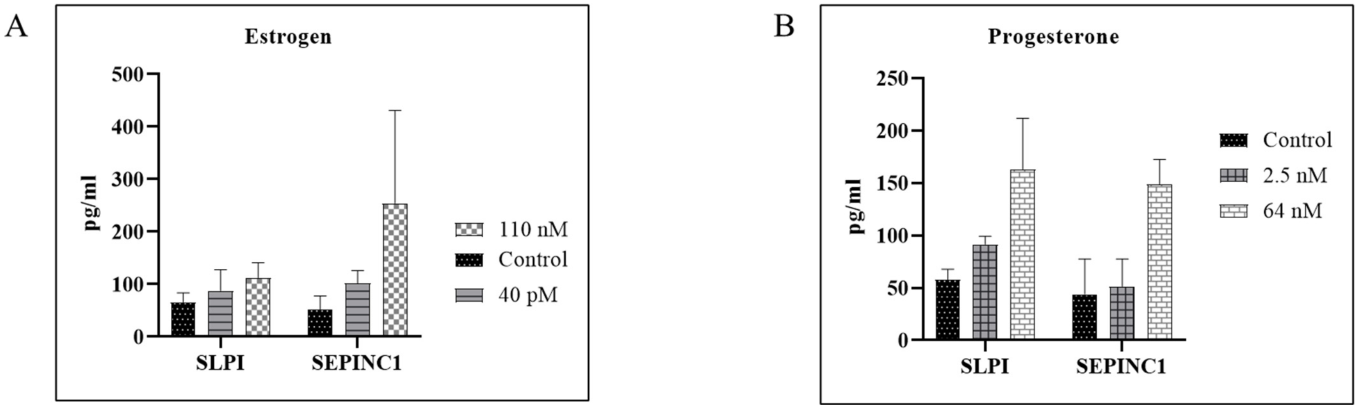

Figure S3: Validation of Protein expression in Estrogen or Progesterone Treated MDMs Infected with HIV-1 (Donor 3).
(A). BioPlex analysis of secreted Secretory Leukocyte Peptidase Inhibitor (SLPI) and Serine Proteinase Inhibitor Clade C Member 1 (SERPIN C1) in culture supernatants from monocytes derived macrophage cells infected with HIV-1 (BaL) pre-treated with 40 pM or 110 nM estrogen (A) or 2.5 nM or 64 nM progesterone (B) and not pre-treated with estrogen or progesterone as control. Culture supernatants were analyzed in duplicate.
Culture supernatants were analyzed in triplicate. Results expressed as mean ± SEM of three independent experimental set up of Donor 3

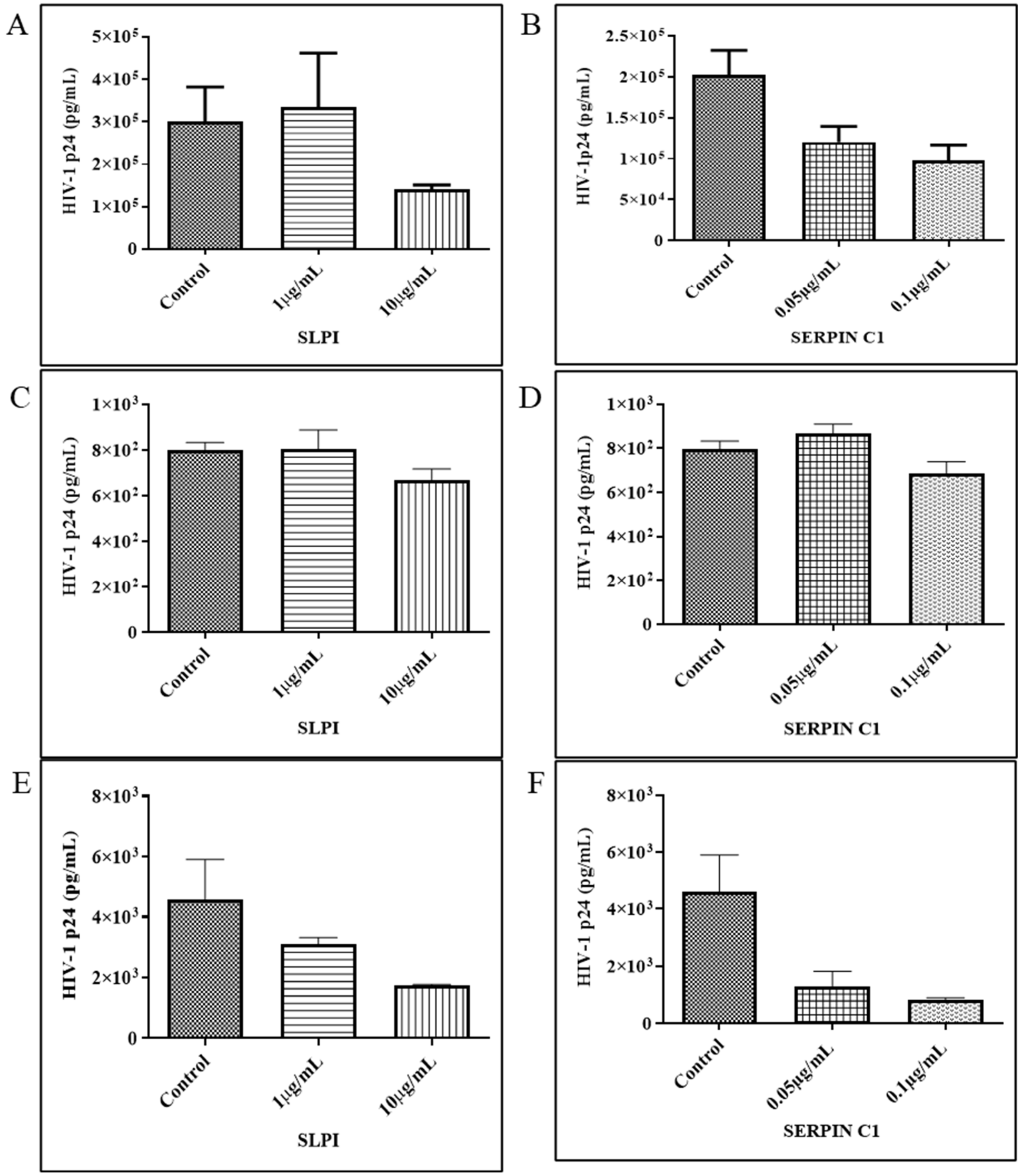

Figure S4: HIV-1 Replication in Pre-treated MDMs (A & B), Post HIV-1 infected MDMs (C & D) and -MDMs infected with pre-incubated inoculum- (E & F) treated with SLPI and SERPINC1 (Donor 2).

(A&B) MDMs (1x106 cells/well) were pre-treated with (A) SLPI 1µg/ml and 10µg/ml or (B) SERPIN C1 0.05µg/ml and 0.1µg/ml concentration for 3 hours then infected with HIV-1 BaL 5 ng/ml p24 units. After 2 hours the virus was removed, and fresh culture media added with 1µg/ml and 10µg/ml SLPI or 0.05µg/ml and 0.1µg/ml SERPINC1 and cultured. (C&D) MDMs (1x106 cells/well) were infected with HIV-1 BaL 5 ng/ml p24 units. After 2 hours the virus was removed, and fresh culture media added with (C) SLPI 1µg/ml and 10µg/ml concentration or (D) SERPIN C1 0.05µg/ml and 0.1µg/ml and cultured the MDMs. (E&F) HIV-1 BaL (5 ng/ml p24 units) was incubated without or with (E) SLPI 1µg/ml and 10µg/ml or (F) SERPIN C1 0.05µg/ml and 0.1µg/ml at 37 °C for 2 h in PBS containing 0.1% BSA. After that, MDMs (5x105 cells/well) were infected with HIV-1 BaL and incubated at 37 °C for 2 h for virus adsorption washed with phosphate buffered saline (PBS) and cultured in 2 ml fresh media at 37 °C.
Culture supernatants were collected after 7 days post infection and HIV-1 replication quantitated by HIV-1 p24 ELISA. Culture supernatants were analyzed in triplicate. Results expressed as mean ± SEM of three independent experimental set up of Donor 2

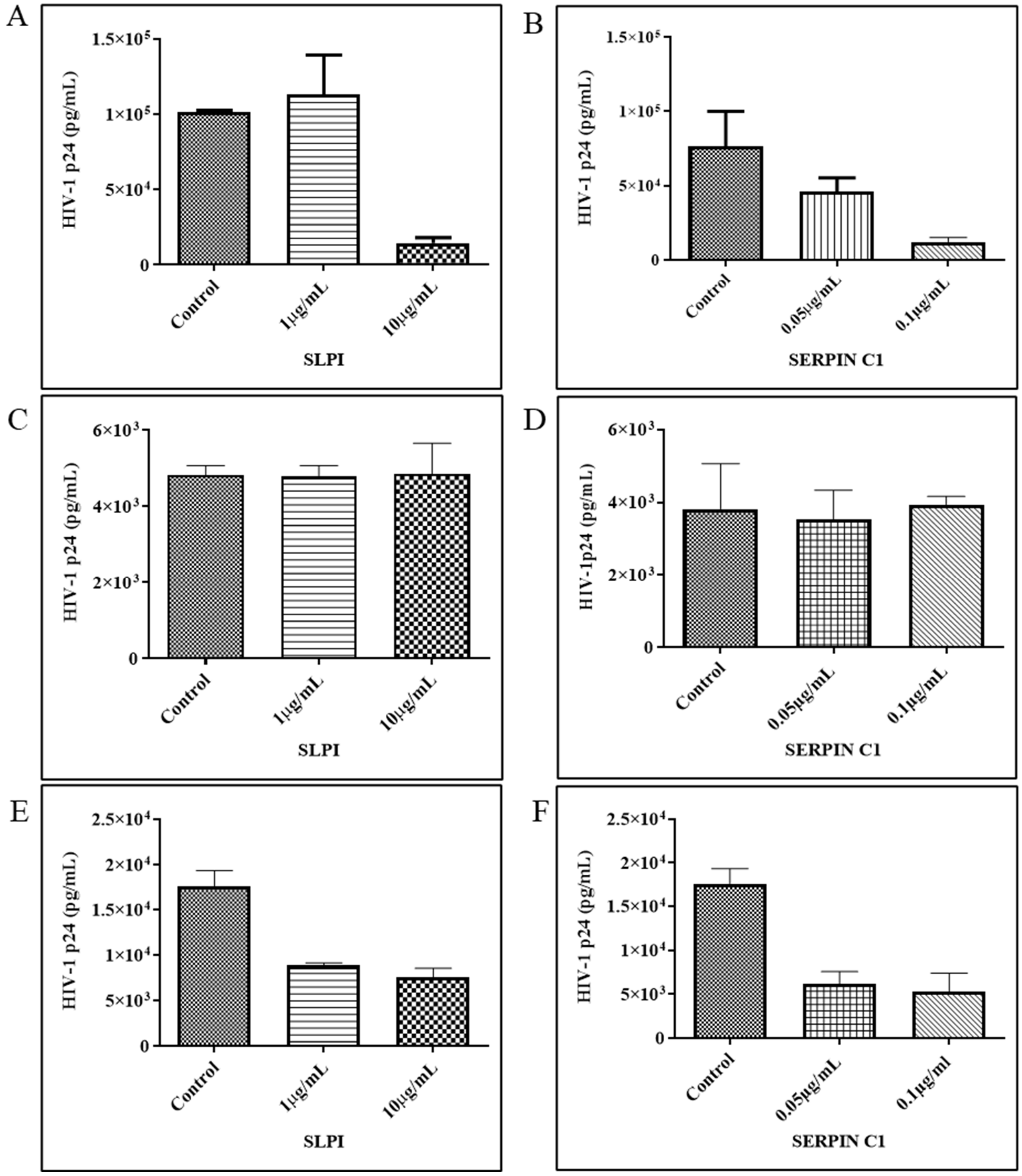

Figure S5: HIV-1 Replication in Pre-treated MDMs (A & B), Post HIV-1 infected MDMs (C & D) and -MDMs infected with pre-incubated inoculum- (E & F) treated with SLPI and SERPINC1 (Donor 3).

(A&B) MDMs (1x106 cells/well) were pre-treated with (A) SLPI 1μg/ml and 10μg/ml or (B) SERPIN C1 0.05μg/ml and 0.1μg/ml concentration for 3 hours then infected with HIV-1 BaL 5 ng/ml p24 units. After 2 hours the virus was removed, and fresh culture media added with 1μg/ml and 10μg/ml SLPI or 0.05μg/ml and 0.1μg/ml SERPINC1 and cultured. (C&D) MDMs (1x106 cells/well) were infected with HIV-1 BaL 5 ng/ml p24 units. After 2 hours the virus was removed, and fresh culture media added with (C) SLPI 1μg/ml and 10μg/ml concentration or (D) SERPIN C1 0.05μg/ml and 0.1μg/ml and cultured the MDMs. (E&F) HIV-1 BaL (5 ng/ml p24 units) was incubated without or with (E) SLPI 1μg/ml and 10μg/ml or (F) SERPIN C1 0.05μg/ml and 0.1μg/ml at 37 °C for 2 h in PBS containing 0.1% BSA. After that, MDMs (5x105 cells/well) were infected with HIV-1 BaL and incubated at 37 °C for 2 h for virus adsorption washed with phosphate buffered saline (PBS) and cultured in 2 ml fresh media at 37 °C.
Culture supernatants were collected after 7 days post infection and HIV-1 replication quantitated by HIV-1 p24 ELISA. Culture supernatants were analyzed in triplicate. Results expressed as mean ± SEM of three independent experimental set up of Donor 3.
